# Methyl 2-benzyl-4-hy­droxy-1,1-dioxo-1,2,3,4-tetra­hydro-1λ^6^,2-benzothia­zine-3-carboxyl­ate

**DOI:** 10.1107/S1600536811020289

**Published:** 2011-06-04

**Authors:** Muhammad Nadeem Arshad, Islam Ullah Khan, Muhammad Zia-ur-Rehman, Muhammad Shafiq, Abdullah M. Asiri

**Affiliations:** a X-ray Diffraction and Crystallography Laboratory, Department of Physics, School of Physical Sciences, University of the Punjab, Quaid-e-Azam Campus, Lahore 54590, Pakistan; b Materials Chemistry Laboratory, Department of Chemistry, GC University, Lahore 54000, Pakistan; c Applied Chemistry Research Centre, PCSIR Laboratories Complex, Lahore 54600, Pakistan; d The Center of Excellence for Advanced Materials Research, King Abdul Aziz University, Jeddah, PO Box 80203, Saudi Arabia

## Abstract

In the title compound, C_17_H_15_NO_5_S, the benzene ring of the fused-ring system is twisted by 11.67 (6)° with respect to the thia­zine ring. The atoms of the four-atom methyl ester group and the phenyl ring of the benzyl unit are inclined at 16.50 (7) and 44.52 (3)° with respect to the thia­zine ring. An intra­molecular O—H⋯O hydrogen bond gives rise to a six-membered *S*(6) ring motif. In the crystal, mol­ecules are extended through a C—H⋯O inter­action along the *a* axis. C—H⋯π inter­actions are also observed.

## Related literature

For the biological properties of benzothia­zines, see: Zia-ur-Rehman *et al.* (2005 ▶, 2006 ▶). For a related structure, see: Arshad *et al.* (2009 ▶). For graph-set notation, see: Bernstein *et al.* (1995 ▶).
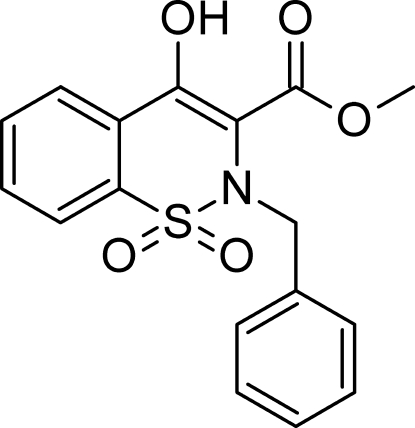


## Experimental

### 

#### Crystal data

C_17_H_15_NO_5_S
*M* *_r_* = 345.36
Monoclinic, 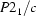

*a* = 9.4920 (15) Å
*b* = 10.9607 (17) Å
*c* = 15.050 (2) Å
β = 99.758 (2)°
*V* = 1543.1 (4) Å^3^
*Z* = 4
Mo *K*α radiation
μ = 0.24 mm^−1^
*T* = 173 K
0.43 × 0.25 × 0.19 mm

#### Data collection

Bruker SMART 1K diffractometer
Absorption correction: multi-scan (*SADABS*; Bruker, 2001 ▶) *T*_min_ = 0.905, *T*_max_ = 0.956
13430 measured reflections
3719 independent reflections
3297 reflections with *I* > 2σ(*I*)
*R*_int_ = 0.033

#### Refinement

*R*[*F* ^2^ > 2σ(*F* ^2^)] = 0.042
*wR*(*F* ^2^) = 0.119
*S* = 1.06
3719 reflections
221 parameters
H atoms treated by a mixture of independent and constrained refinement
Δρ_max_ = 0.79 e Å^−3^
Δρ_min_ = −0.57 e Å^−3^

### 

Data collection: *SMART* (Bruker, 2001 ▶); cell refinement: *SAINT* (Bruker, 2001 ▶); data reduction: *SAINT*; program(s) used to solve structure: *SHELXS97* (Sheldrick, 2008 ▶); program(s) used to refine structure: *SHELXL97* (Sheldrick, 2008 ▶); molecular graphics: *ORTEP-3 for Windows* (Farrugia, 1997 ▶) and *PLATON* (Spek, 2009 ▶); software used to prepare material for publication: *X-SEED* (Barbour, 2001) ▶, *WinGX* (Farrugia, 1999 ▶) and *PLATON*.

## Supplementary Material

Crystal structure: contains datablock(s) I, global. DOI: 10.1107/S1600536811020289/ng5172sup1.cif

Structure factors: contains datablock(s) I. DOI: 10.1107/S1600536811020289/ng5172Isup2.hkl

Supplementary material file. DOI: 10.1107/S1600536811020289/ng5172Isup3.cml

Additional supplementary materials:  crystallographic information; 3D view; checkCIF report

## Figures and Tables

**Table 1:** Hydrogen-bond geometry (Å, °) *Cg*1 is the centroid of the C1–C6 ring.

*D*—H⋯*A*	*D*—H	H⋯*A*	*D*⋯*A*	*D*—H⋯*A*
C2—H2⋯O4^i^	0.95	2.49	3.1861 (19)	130
O1—H1*O*⋯O4	0.93 (2)	1.72 (2)	2.5580 (15)	149 (2)
C10—H10*B*⋯*Cg*1^ii^	0.80	2.94	3.6391 (18)	130

Symmetry codes: (i) 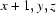; (ii) 
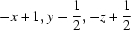
.

**Fig. 1.**
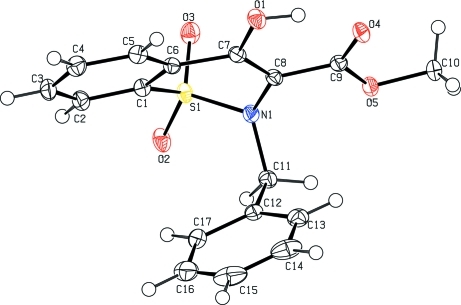
The labelled diagram of (I) with thermal ellipsoids drawn at 50% probability level.

**Fig. 2.**
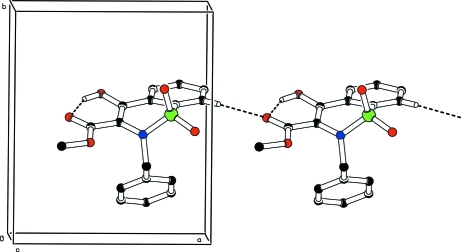
Unit cell packing for (I) showinh the inter and intramolecular hydrogen bondings using dashed lines. Hydrogen atoms not involved in hydrogen bonding have been omitted for clarity.

## supplementary crystallographic information

### Comment

Crystallographic and biological studies of benzothiazine molecules and their derivatives gained much attraction in recent literature (Zia-ur-Rehman *et al.* 2005, 2006) and (Arshad *et al.* 2009).

The title compound is *N*-alkylated derivative of methyl -4-hydroxy-2*H*-1,2-benzothiazine-3-carboxylate 1,1-dioxide. The methyl ester moiety attached to the thiazine ring is almost planer showing the r. m. s. deviation of 0.0087Å and is oriented at dihedral angle of 16.50 (7)° with respect to the thiazine ring. The typical intramolecular O–H···O interaction of 4-hydroxy benzothiazine molecules observed and generates almost planer six membered ring motif S_1_^1^(6)(Bernstein, *et al.*, 1995) with the r.m.s deviaton of 0.0085Å and produces dihedral angles of 16.02 (33)° & 15.87 (32)° with respect to the thiazine and aromatic (C1/C2/C3/C4/C5/C6) rings respectively. The thiazine ring adopted the half chair shaped as the S1 and N1 showed maximum deviation from the least square plane measure 0.3113 (7)Å and 0.3082 (8)Å respectively and root mean square deviation for the ring is 0.2031Å. A weak intermolecular hydrogen bonding interaction observed along the *a* axes (Fig.2. Tab.1). Further hydrogen atom from methyl group of ester moiety involved in symmetry related C—H···.π interaction [C10—H10B···.C*g*1, where C*g*1 is centroid of (C1—C6)] table 1. The benzyl ring attached to nitogen atom of thiazine ring is oriented at dihedral angle of 51.33 (3)° and 44.52 (3)° with  respect to the aromatic (C1—C6) and thiazine rings respectively.

### Experimental

A mixture of methyl 4-hydroxy-2H-1,2-benzothiazine-3-carboxylate-1,1-dioxide (350 mg, 1.37 mmol), sodium hydride (77 mg, 3.2 mmol) and dimethylformamide (5 ml) was prperaed in a round bottom flask. Benzyl Chloride (202 mg, 1.6 mmol) was added drop wise to the above mixture. Contents were allowed to stirr at room temperature for 5 h under nitrogen atmosphere and poured over ice cooled water (100 ml). The pH was adjusted 2-3 using 1N HCL which yielded precipetates . The precipitates were filtered, washed with cold water and dried. Single crystals were obtained by re-crystallization from a methanol solution under slow evaporation.

### Refinement

All the C—H H-atoms were positioned with idealized geometry with C—H = 0.93000 Å for aromatic, C—H = 0.96000 Å for methylene, C—H = 0.97000 Å for methyl and were refined using a riding model with *U*_iso_(H) = 1.2 *U*_eq_for aromatic C atoms. The O—H H-atom was located via fourier map with O—H = 0.93 (2) Å with *U*_iso_(H) = 1.5 *U*_eq_for O atom. The three reflection   -6 2 1, 9 0 2 and -7 1 2 were omitted in final refinement as these were highly obscured by beamstop.

### Crystal data

**Table:**

C_17_H_15_NO_5_S	*F*(000) = 720
*M**_r_* = 345.36	*D*_x_ = 1.487 Mg m^−^^3^
Monoclinic, *P*2_1_/*c*	Melting point: 422 K
Hall symbol: -P 2ybc	Mo *K*α radiation, λ = 0.71073 Å
*a* = 9.4920 (15) Å	Cell parameters from 6007 reflections
*b* = 10.9607 (17) Å	θ = 2.3–28.3°
*c* = 15.050 (2) Å	µ = 0.24 mm^−^^1^
β = 99.758 (2)°	*T* = 173 K
*V* = 1543.1 (4) Å^3^	Block, colorless
*Z* = 4	0.43 × 0.25 × 0.19 mm

### Data collection

**Table:**

Bruker SMART 1K diffractometer	3719 independent reflections
Radiation source: fine-focus sealed tube	3297 reflections with *I* > 2σ(*I*)
graphite	*R*_int_ = 0.033
φ and ω scans	θ_max_ = 28.4°, θ_min_ = 2.2°
Absorption correction: multi-scan (*SADABS*; Bruker, 2007)	*h* = −12→12
*T*_min_ = 0.905, *T*_max_ = 0.956	*k* = −14→14
13430 measured reflections	*l* = −20→19

### Refinement

**Table:**

Refinement on *F*^2^	Primary atom site location: structure-invariant direct methods
Least-squares matrix: full	Secondary atom site location: difference Fourier map
*R*[*F*^2^ > 2σ(*F*^2^)] = 0.042	Hydrogen site location: inferred from neighbouring sites
*wR*(*F*^2^) = 0.119	H atoms treated by a mixture of independent and constrained refinement
*S* = 1.06	*w* = 1/[σ^2^(*F*_o_^2^) + (0.0721*P*)^2^ + 0.6338*P*] where *P* = (*F*_o_^2^ + 2*F*_c_^2^)/3
3719 reflections	(Δ/σ)_max_ = 0.001
221 parameters	Δρ_max_ = 0.79 e Å^−^^3^
0 restraints	Δρ_min_ = −0.57 e Å^−^^3^

### Special details

**Table:**

Geometry. All s.u.'s (except the s.u. in the dihedral angle between two l.s. planes) are estimated using the full covariance matrix. The cell s.u.'s are taken into account individually in the estimation of s.u.'s in distances, angles and torsion angles; correlations between s.u.'s in cell parameters are only used when they are defined by crystal symmetry. An approximate (isotropic) treatment of cell s.u.'s is used for estimating s.u.'s involving l.s. planes.
Refinement. Refinement of F^2^ against ALL reflections. The weighted R-factor wR and goodness of fit S are based on F^2^, conventional R-factors R are based on F, with F set to zero for negative F^2^. The threshold expression of F^2^ > 2σ(F^2^) is used only for calculating R-factors(gt) etc. and is not relevant to the choice of reflections for refinement. R-factors based on F^2^ are statistically about twice as large as those based on F, and R- factors based on ALL data will be even larger.

### Fractional atomic coordinates and isotropic or equivalent isotropic displacement parameters (Å^2^)

**Table:**

	*x*	*y*	*z*	*U*_iso_*/*U*_eq_	
S1	0.81488 (4)	0.52594 (3)	0.28570 (2)	0.01595 (12)	
O4	0.30241 (11)	0.50771 (10)	0.17813 (7)	0.0183 (2)	
C1	0.83777 (15)	0.56206 (13)	0.17521 (10)	0.0157 (3)	
O1	0.46409 (11)	0.60454 (10)	0.07788 (7)	0.0187 (2)	
C2	0.97337 (16)	0.58108 (14)	0.15447 (10)	0.0188 (3)	
H2	1.0565	0.5666	0.1981	0.023*	
O5	0.40378 (11)	0.40446 (10)	0.30203 (7)	0.0177 (2)	
C3	0.98459 (17)	0.62175 (14)	0.06835 (11)	0.0209 (3)	
H3	1.0763	0.6351	0.0529	0.025*	
O2	0.93353 (11)	0.45544 (11)	0.33020 (7)	0.0220 (3)	
C4	0.86248 (17)	0.64307 (14)	0.00470 (10)	0.0204 (3)	
H4	0.8715	0.6726	−0.0534	0.025*	
O3	0.77720 (12)	0.63674 (10)	0.32661 (7)	0.0211 (2)	
C5	0.72758 (16)	0.62151 (13)	0.02546 (10)	0.0176 (3)	
H5	0.6448	0.6353	−0.0185	0.021*	
N1	0.67339 (13)	0.43784 (11)	0.26853 (8)	0.0156 (3)	
C6	0.71401 (15)	0.57923 (13)	0.11162 (10)	0.0152 (3)	
C7	0.57231 (15)	0.55402 (13)	0.13462 (10)	0.0153 (3)	
C8	0.55400 (15)	0.48728 (13)	0.20867 (10)	0.0150 (3)	
C9	0.40936 (16)	0.46801 (13)	0.22778 (10)	0.0154 (3)	
C11	0.69172 (16)	0.30226 (13)	0.26997 (10)	0.0169 (3)	
H11A	0.6125	0.2651	0.2957	0.020*	
H11B	0.7821	0.2818	0.3105	0.020*	
C12	0.69472 (16)	0.24606 (13)	0.17890 (10)	0.0165 (3)	
C13	0.56919 (17)	0.20116 (14)	0.12780 (11)	0.0212 (3)	
H13	0.4816	0.2081	0.1500	0.025*	
C14	0.5710 (2)	0.14639 (16)	0.04476 (12)	0.0307 (4)	
H14	0.4848	0.1168	0.0101	0.037*	
C15	0.6992 (2)	0.13497 (16)	0.01245 (12)	0.0340 (4)	
H15	0.7007	0.0970	−0.0441	0.041*	
C16	0.8247 (2)	0.17886 (16)	0.06271 (12)	0.0308 (4)	
H16	0.9123	0.1703	0.0408	0.037*	
C10	0.26012 (16)	0.38959 (15)	0.32309 (11)	0.0199 (3)	
H10A	0.2184	0.4700	0.3303	0.030*	
H10B	0.2652	0.3431	0.3792	0.030*	
H10C	0.2003	0.3456	0.2739	0.030*	
C17	0.82300 (18)	0.23540 (14)	0.14515 (11)	0.0225 (3)	
H17	0.9091	0.2669	0.1787	0.027*	
H1O	0.381 (3)	0.583 (2)	0.0989 (14)	0.034*	

### Atomic displacement parameters (Å^2^)

**Table:**

	*U*^11^	*U*^22^	*U*^33^	*U*^12^	*U*^13^	*U*^23^
S1	0.01167 (19)	0.0206 (2)	0.0152 (2)	−0.00077 (12)	0.00123 (13)	0.00070 (13)
O4	0.0117 (5)	0.0229 (5)	0.0201 (5)	0.0010 (4)	0.0018 (4)	−0.0008 (4)
C1	0.0153 (7)	0.0163 (7)	0.0155 (7)	0.0000 (5)	0.0030 (5)	0.0000 (5)
O1	0.0134 (5)	0.0227 (6)	0.0193 (5)	0.0025 (4)	0.0007 (4)	0.0035 (4)
C2	0.0148 (7)	0.0206 (7)	0.0210 (7)	0.0001 (5)	0.0030 (5)	0.0004 (6)
O5	0.0134 (5)	0.0198 (5)	0.0207 (5)	0.0004 (4)	0.0051 (4)	0.0023 (4)
C3	0.0174 (7)	0.0230 (8)	0.0240 (8)	−0.0024 (6)	0.0085 (6)	−0.0010 (6)
O2	0.0121 (5)	0.0308 (6)	0.0219 (6)	0.0010 (4)	−0.0002 (4)	0.0060 (4)
C4	0.0236 (8)	0.0207 (7)	0.0181 (7)	−0.0009 (6)	0.0066 (6)	0.0007 (6)
O3	0.0199 (5)	0.0238 (6)	0.0196 (5)	−0.0038 (4)	0.0033 (4)	−0.0037 (4)
C5	0.0175 (7)	0.0179 (7)	0.0169 (7)	0.0004 (5)	0.0015 (5)	−0.0009 (5)
N1	0.0122 (6)	0.0165 (6)	0.0178 (6)	0.0004 (4)	0.0018 (5)	0.0015 (5)
C6	0.0147 (7)	0.0142 (7)	0.0169 (7)	0.0005 (5)	0.0030 (5)	−0.0012 (5)
C7	0.0136 (7)	0.0152 (6)	0.0169 (7)	0.0013 (5)	0.0018 (5)	−0.0021 (5)
C8	0.0119 (7)	0.0149 (7)	0.0176 (7)	0.0016 (5)	0.0010 (5)	−0.0008 (5)
C9	0.0153 (7)	0.0137 (7)	0.0172 (7)	−0.0004 (5)	0.0028 (5)	−0.0028 (5)
C11	0.0161 (7)	0.0174 (7)	0.0173 (7)	0.0025 (5)	0.0028 (5)	0.0033 (5)
C12	0.0178 (7)	0.0134 (7)	0.0186 (7)	0.0017 (5)	0.0039 (5)	0.0031 (5)
C13	0.0195 (7)	0.0174 (7)	0.0250 (8)	0.0022 (6)	−0.0012 (6)	0.0029 (6)
C14	0.0420 (10)	0.0189 (8)	0.0259 (8)	0.0036 (7)	−0.0092 (7)	0.0020 (6)
C15	0.0651 (13)	0.0198 (8)	0.0181 (8)	0.0076 (8)	0.0103 (8)	0.0023 (6)
C16	0.0448 (11)	0.0209 (8)	0.0330 (9)	0.0054 (7)	0.0244 (8)	0.0064 (7)
C10	0.0140 (7)	0.0219 (8)	0.0249 (8)	−0.0017 (5)	0.0066 (6)	0.0006 (6)
C17	0.0228 (8)	0.0185 (7)	0.0286 (8)	−0.0006 (6)	0.0110 (6)	0.0033 (6)

### Geometric parameters (Å, °)

**Table:**

S1—O3	1.4341 (12)	C6—C7	1.471 (2)
S1—O2	1.4347 (11)	C7—C8	1.369 (2)
S1—N1	1.6387 (13)	C8—C9	1.465 (2)
S1—C1	1.7583 (15)	C11—C12	1.507 (2)
O4—C9	1.2333 (18)	C11—H11A	0.9900
C1—C2	1.391 (2)	C11—H11B	0.9900
C1—C6	1.397 (2)	C12—C13	1.394 (2)
O1—C7	1.3393 (17)	C12—C17	1.401 (2)
O1—H1O	0.93 (2)	C13—C14	1.389 (2)
C2—C3	1.392 (2)	C13—H13	0.9500
C2—H2	0.9500	C14—C15	1.391 (3)
O5—C9	1.3254 (18)	C14—H14	0.9500
O5—C10	1.4606 (17)	C15—C16	1.384 (3)
C3—C4	1.393 (2)	C15—H15	0.9500
C3—H3	0.9500	C16—C17	1.389 (2)
C4—C5	1.389 (2)	C16—H16	0.9500
C4—H4	0.9500	C10—H10A	0.9800
C5—C6	1.403 (2)	C10—H10B	0.9800
C5—H5	0.9500	C10—H10C	0.9800
N1—C8	1.4295 (18)	C17—H17	0.9500
N1—C11	1.4959 (19)		
			
O3—S1—O2	119.27 (7)	N1—C8—C9	119.40 (13)
O3—S1—N1	108.02 (6)	O4—C9—O5	123.35 (13)
O2—S1—N1	108.29 (7)	O4—C9—C8	122.20 (13)
O3—S1—C1	107.16 (7)	O5—C9—C8	114.46 (13)
O2—S1—C1	110.47 (7)	N1—C11—C12	114.38 (12)
N1—S1—C1	102.29 (7)	N1—C11—H11A	108.7
C2—C1—C6	121.94 (13)	C12—C11—H11A	108.7
C2—C1—S1	120.90 (11)	N1—C11—H11B	108.7
C6—C1—S1	117.00 (11)	C12—C11—H11B	108.7
C7—O1—H1O	106.1 (14)	H11A—C11—H11B	107.6
C1—C2—C3	118.48 (14)	C13—C12—C17	118.98 (14)
C1—C2—H2	120.8	C13—C12—C11	119.99 (13)
C3—C2—H2	120.8	C17—C12—C11	121.01 (14)
C9—O5—C10	114.45 (11)	C14—C13—C12	120.60 (15)
C2—C3—C4	120.55 (14)	C14—C13—H13	119.7
C2—C3—H3	119.7	C12—C13—H13	119.7
C4—C3—H3	119.7	C13—C14—C15	119.92 (17)
C5—C4—C3	120.55 (14)	C13—C14—H14	120.0
C5—C4—H4	119.7	C15—C14—H14	120.0
C3—C4—H4	119.7	C16—C15—C14	120.03 (16)
C4—C5—C6	119.77 (14)	C16—C15—H15	120.0
C4—C5—H5	120.1	C14—C15—H15	120.0
C6—C5—H5	120.1	C15—C16—C17	120.24 (16)
C8—N1—C11	117.62 (11)	C15—C16—H16	119.9
C8—N1—S1	114.65 (10)	C17—C16—H16	119.9
C11—N1—S1	119.53 (10)	O5—C10—H10A	109.5
C1—C6—C5	118.65 (13)	O5—C10—H10B	109.5
C1—C6—C7	120.66 (13)	H10A—C10—H10B	109.5
C5—C6—C7	120.68 (13)	O5—C10—H10C	109.5
O1—C7—C8	123.41 (13)	H10A—C10—H10C	109.5
O1—C7—C6	113.92 (12)	H10B—C10—H10C	109.5
C8—C7—C6	122.65 (13)	C16—C17—C12	120.22 (16)
C7—C8—N1	121.28 (13)	C16—C17—H17	119.9
C7—C8—C9	119.31 (13)	C12—C17—H17	119.9
			
O3—S1—C1—C2	98.81 (13)	O1—C7—C8—N1	−178.36 (13)
O2—S1—C1—C2	−32.60 (15)	C6—C7—C8—N1	0.0 (2)
N1—S1—C1—C2	−147.69 (13)	O1—C7—C8—C9	0.5 (2)
O3—S1—C1—C6	−76.69 (13)	C6—C7—C8—C9	178.95 (13)
O2—S1—C1—C6	151.90 (11)	C11—N1—C8—C7	−111.02 (15)
N1—S1—C1—C6	36.80 (13)	S1—N1—C8—C7	37.42 (17)
C6—C1—C2—C3	1.9 (2)	C11—N1—C8—C9	70.08 (17)
S1—C1—C2—C3	−173.42 (11)	S1—N1—C8—C9	−141.49 (11)
C1—C2—C3—C4	0.1 (2)	C10—O5—C9—O4	−2.7 (2)
C2—C3—C4—C5	−1.4 (2)	C10—O5—C9—C8	177.58 (12)
C3—C4—C5—C6	0.7 (2)	C7—C8—C9—O4	1.7 (2)
O3—S1—N1—C8	61.81 (11)	N1—C8—C9—O4	−179.41 (13)
O2—S1—N1—C8	−167.73 (10)	C7—C8—C9—O5	−178.62 (13)
C1—S1—N1—C8	−51.05 (11)	N1—C8—C9—O5	0.31 (19)
O3—S1—N1—C11	−150.40 (10)	C8—N1—C11—C12	54.00 (17)
O2—S1—N1—C11	−19.94 (13)	S1—N1—C11—C12	−92.85 (14)
C1—S1—N1—C11	96.74 (11)	N1—C11—C12—C13	−93.63 (16)
C2—C1—C6—C5	−2.5 (2)	N1—C11—C12—C17	87.87 (17)
S1—C1—C6—C5	172.92 (11)	C17—C12—C13—C14	0.1 (2)
C2—C1—C6—C7	177.73 (14)	C11—C12—C13—C14	−178.38 (14)
S1—C1—C6—C7	−6.82 (19)	C12—C13—C14—C15	0.6 (2)
C4—C5—C6—C1	1.2 (2)	C13—C14—C15—C16	−0.4 (3)
C4—C5—C6—C7	−179.06 (14)	C14—C15—C16—C17	−0.6 (3)
C1—C6—C7—O1	162.79 (13)	C15—C16—C17—C12	1.4 (2)
C5—C6—C7—O1	−16.94 (19)	C13—C12—C17—C16	−1.2 (2)
C1—C6—C7—C8	−15.8 (2)	C11—C12—C17—C16	177.36 (14)
C5—C6—C7—C8	164.52 (14)		

### Hydrogen-bond geometry (Å, °)

**Table:**

Cg1 is the centroid of the C1–C6 ring.

**Table:**

*D*—H···*A*	*D*—H	H···*A*	*D*···*A*	*D*—H···*A*
C2—H2···O4^i^	0.95	2.49	3.1861 (19)	130
O1—H1O···O4	0.93 (2)	1.72 (2)	2.5580 (15)	149 (2)
C10—H10B···Cg1^ii^	0.80	2.94	3.6391 (18)	130

Symmetry codes: (i) *x*+1, *y*, *z*; (ii) −*x*+1, *y*−1/2, −*z*+1/2.
